# Use of annual surveying to identify technology trends and improve service provision

**DOI:** 10.5195/jmla.2018.324

**Published:** 2018-07-01

**Authors:** Hannah F. Norton, Michele R. Tennant, Mary E. Edwards, Ariel Pomputius

**Affiliations:** Reference and Liaison Librarian, University of Florida Health Science Center Libraries, Gainesville, FL; Associate Director, University of Florida Health Science Center Libraries, Gainesville, FL; Reference and Liaison Librarian, University of Florida Health Science Center Libraries, Gainesville, FL; Health Sciences Liaison Librarian, Biomedical and Health Information Services, University of Florida Health Science Center Libraries, Gainesville, FL

## Abstract

**Objective:**

At an academic health sciences library serving a wide variety of disciplines, studying library users’ technology use provides necessary information on intersection points for library services. Administering a similar survey annually for five years generated a holistic view of users’ technology needs and preferences over time.

**Methods:**

From 2012 to 2016, the University of Florida Health Science Center Library (HSCL) annually administered a sixteen-to-twenty question survey addressing health sciences users’ technology awareness and use and their interest in using technology to engage with the library and its services. The survey was distributed throughout the HSC via email invitation from liaison librarians to their colleges and departments and advertisement on the HSCL home page.

**Results:**

Smartphone ownership among survey respondents was nearly universal, and a majority of respondents also owned a tablet. While respondents were likely to check library hours, use medical apps, and use library electronic resources from their mobile devices, they were unlikely to friend or follow the library on Facebook or Twitter or send a call number from the catalog. Respondents were more likely to have used EndNote than any other citation management tool, but over 50% of respondents had never used each tool or never heard of it.

**Conclusions:**

Annual review of survey results has allowed librarians to identify users’ needs and interests, leading to incremental changes in services offered. Reviewing the aggregate data allowed strategic consideration of how technology impacts library interactions with users, with implications toward library marketing, training, and service development.

## INTRODUCTION

As technology continues to develop, health sciences information professionals have a responsibility to stay up-to-date on emerging and existing technologies that their communities are using in order to adapt their programs and offerings to best support those communities. To that end, the Health Science Center Library (HSCL) at the University of Florida (UF) has administered an annual technology survey to assess how library patrons prefer to use their mobile devices to interact with the library and what types of technologies they use. Faculty, staff, and students in UF’s six Health Science Center (HSC) colleges (Dentistry, Medicine, Nursing, Pharmacy, Public Health & Health Professions, and Veterinary Medicine) participated in the survey.

Prior to this effort, the HSCL had conducted an initial foray into studying the technology habits of library users [1]. Results from that smartphone-focused survey demonstrated that library users were using these tools and other mobile devices for a variety of purposes, including supporting clinical, research, and instructional duties. The results of that survey suggested that additional investigation in this area would be useful to the HSCL, given the changing mobile technology landscape.

Assessing patrons’ technology usage is common in health sciences libraries, but typically those studies focus on the information resources available on new devices and the role that the library should take regarding emerging technology. For instance, in 2012, the staff at the Dana Medical Library at the University of Vermont provided services and instruction on clinical medical apps for third-year medical students. In a post-program evaluation, students reported that having a mobile device on their rotation improved their access to information and overall clinical experience and that library support was valuable, despite the fact that the two most-used applications did not require a library subscription [2]. Mi et al. conducted a systematic review to better understand the types of information resources accessed from these devices and the benefit for clinical diagnosis, evidence-based practice support, and student learning [3]. A 2012 survey created by the University of Southern California (USC) Health Sciences Libraries staff concluded that first-year medical students owned multiple technological devices but were often unaware of newer technology and urged librarians to develop educational workshops to fill knowledge gaps regarding emerging technologies [4].

While librarians are interested in how libraries can support clinicians using mobile apps, the broader health sciences community has also delved into the question of how students and clinicians use emerging technologies. In 2012, Mickan et al. reported that mobile devices were effective in patient care and evidence-based practice [5]. Other studies reported similar findings: that medical students and clinicians were increasingly using mobile technologies in clinical settings and find them useful [6, 7]. More recently, Sandholzer et al. found that the greatest factors impacting the adoption of a particular mobile application by medical students included perceived benefit of use, personal interest in new technologies, perceived impact of previously adopted mobile technologies, and gender [8]. With more students and residents identifying as early adopters, medical education programs and libraries are encouraging the use of emerging devices in the clinics and the classroom and supporting new technology with appropriate instruction and infrastructure [9].

As the use of technology in health care evolves and libraries strive to serve users in all of their information needs, evaluating users’ technological habits is important to understanding how they engage with library resources to accomplish their goals. The purposes of this study are to analyze longitudinal data from UF HSCL users about their technology needs and preferences, identify changes over time, highlight trends that may be generalizable to a broad health sciences library population versus those that are locally specific, and use these results to inform technology acquisition, policy, and training at UF HSCL.

## METHODS

Beginning in 2012, HSCL librarians annually administered a survey designed by the USC Health Sciences Libraries to address health sciences students’ and faculty’s awareness and use of technology, as well as their interest in using technology to engage with the library and its services [4]. While USC’s survey was targeted initially toward first-year students only, HSCL’s implementation was distributed to all HSC students, faculty, and staff.

For three years, the HSCL participated in a multi-institutional implementation of this survey led by USC; when the collaboration ended, the HSCL team continued to administer the survey at UF. During the course of the multi-institutional collaboration, each institution was able to include several unique questions, but all other questions and response options were uniform across institutions. While some questions have been modified over time for clarity or changes in available technology, most are consistent across the five years of survey implementation.

In the fall of each year, HSCL librarians sought and obtained institutional review board (IRB-02) exemption for the current version of the survey and distributed the survey throughout the HSC via email invitation from liaison librarians to their colleges and departments, as well as advertisement on the HSCL home page. The survey was created and administered electronically in either UF’s SurveyMonkey account (2012), UF’s Qualtrics account (2013, 2015, 2016), or USC’s Qualtrics account (2014). Over the years, the survey grew from sixteen to twenty questions, and it was kept open for four to twelve weeks each year. The 2016 version of the survey is available in supplemental Appendix A.

## RESULTS

The number of responses remained relatively consistent over the past 5 years, with a distinct decrease in 2014 and increase in 2016. Total numbers of responses each year, including only those who answered at least one question after consenting to participate, were as follows: 268 in 2012, 289 in 2013, 215 in 2014, 290 in 2015, and 351 in 2016; the overall response rate over 5 years was 1.9%. Responses came mostly from students in the HSC’s professional programs (39.0% of responses over all 5 years) and graduate programs (23.2%), as well as from faculty (20.1%), staff (11.1%), undergraduate students (3.8%), residents (1.3%), postdoctoral associates (1.0%), and other community members (1.1%). While temporal trends were examined for each question, for the most part, there were not significant changes over time. Exceptions are mentioned explicitly below, and, unless otherwise noted, results below report on the aggregate responses across all 5 years of data collection. A summary of all statistical tests and results, as conducted using SPSS statistical software, is available in supplementary Appendix B.

A series of questions asked respondents about their technology use, both in terms of equipment or hardware and social sites. Respondents were first asked what kind of technology they used. Responses were smartphones (92.8%), tablets (61.7%), PC laptops (61.5%), PC desktop computers (44.2%), Mac laptops (40.8%), wearable technology (23.7%), e-book readers (22.0%), Mac desktop computers (9.1%), virtual reality hardware (0.9%), and other (0.1%). Only smartphones and tablets showed a significant increase in use over time (analysis of variance ([ANOVA], *p*<0.01), although wearable technology and virtual reality hardware were only included as options in 2016.

**Figure 1:**
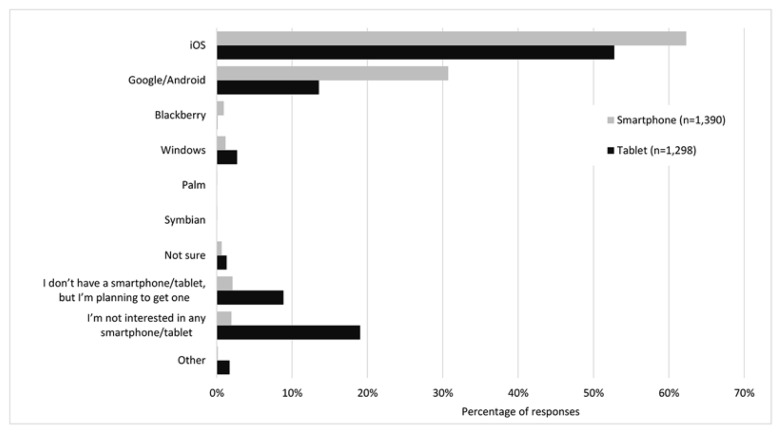
Operating systems of smartphones and tablets

Likewise, respondents were asked which operating systems they had on their smartphone and tablet (Figure 1). There was a significant decline in the use of the Blackberry smartphone operating system over time (ANOVA, *p*<0.01), as well as a decline in respondents who were unsure of their smartphone operating system (*p*<0.01), did not have a smartphone but were planning to get one (*p*<0.01), or reported being uninterested in any smartphone (*p*<0.01). For tablets, there was also a significant increase in use of the Google/Android (*p*<0.05) and Windows (*p*<0.05) operating systems over time.

**Figure 2:**
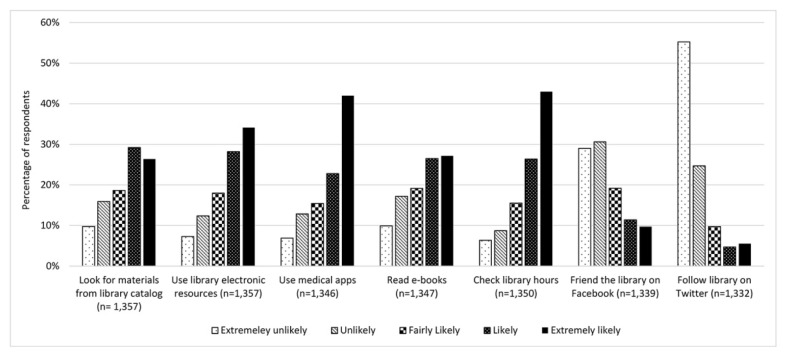
Likelihood of smartphone or tablet owners using library services

Respondents were asked to rate how likely they would be to use a series of library services on their smartphone or tablet on a scale of 1 (extremely unlikely) to 5 (extremely likely) (Figure 2). The most popular responses were check library hours (average score of 3.91), use medical apps (3.80), and use library electronic resources (3.70). Less popular responses were follow the library on Twitter (1.81) and friend the library on Facebook (2.42). There was a significant increase over time in the likelihood of checking library hours (ANOVA, *p*<0.05) and following the library on Twitter (*p*<0.05). There was a significant decrease over time in the likelihood of using library electronic resources (*p*<0.01) and using medical apps (*p*<0.01). Similarly, respondents were asked to rate how likely they would be to use a series text/SMS library services on a scale of 1 (extremely unlikely) to 5 (extremely likely). Overall, respondents were more likely to use 2 library services—receive renewal or overdue notices (3.42) and renew library materials (3.40)—and were less likely to use 2 other library services—send a call number from the catalog (2.65) and ask a librarian a question (2.68).

**Figure 3:**
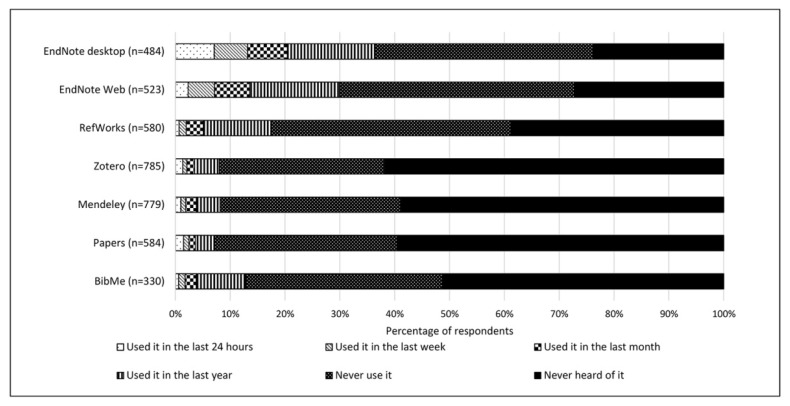
Usage of citation tools

In part to gauge which citation management software the library should support, the survey asked respondents to indicate their usage of specific tools: whether they had used them in the past 24 hours, week, month, or year; had never used them; or had never heard of them (Figure 3). The most frequently used tools were EndNote (desktop), EndNote Web, and RefWorks. Over 50% of respondents had not heard of the other listed tools: Zotero, Mendeley, Papers, and BibMe. Additionally, responses were analyzed to determine whether new students were less likely to be familiar with citation management tools than returning students. Significantly more new student respondents (28.2%) than returning students (17.4%) indicated that they had not heard of any of the citation management tools listed in the survey (chi-square, *p*<0.05).

The survey included 2 questions regarding respondents’ preferences for e-books or print books, both for academic purposes and for leisure reading. Respondents preferred reading print books (55.2%) over e-books (23.6%) for academic purposes, although several individuals had no preference (21.2%). Respondents also preferred reading print books (53.8%) over e-books (23.7%) for leisure purposes, with several respondents having no preference (22.4%).

One of the questions that was customized for UF asked respondents what, if any, training topics they were interested in (Table 1). Initially, the options were focused primarily around mobile apps and other technology-centric topics. Over time, the HSCL team chose to include additional information-centric topics, some of which were already being taught by HSCL librarians. Some of the most popular responses were: clinical mobile device apps (52.8%), photo editing tools (42.7%), presentation tools (43.0%), mobile device apps for research (41.3%), and mobile device apps for productivity (40.4%). Training interest results were analyzed by the colleges of the respondents for future use by HSCL liaison librarians in developing resources for specific colleges or programs (data not shown).

**Table 1:** Interest in topics for training

Training topic	Number of responses					

	2012	2013	2014	2015	2016	Total
Clinical mobile device apps	127	136	76	114	137	590
Photo editing tools	65	96	84	100	143	488
Presentation tools (PowerPoint, Prezi)	73	109	66	87	145	480
Google tools	72	81	64	99	139	455
Mobile device apps for research	100	106	NA	114	141	461
Mobile device apps for productivity	99	101	69	103	148	451
Mobile device apps for education	106	102	67	95	124	427
Citation tools	NA	NA	88	129	172	389
Database searching	NA	NA	98	111	153	362
Video editing tools	54	66	61	74	107	362
Keeping up with current research	NA	NA	74	104	132	310
Mobile device apps for a particular platform (iPhone, iPad, Android devices)	96	92	NA	NA	NA	188
3D printing	NA	NA	NA	81	102	183
Patient-oriented apps	NA	NA	NA	NA	118	118
Social networking tools	25	36	24	NA	NA	85
Virtual reality hardware	NA	NA	NA	NA	56	56
Other	17	12	8	2	5	44
Blogs	19	17	NA	NA	NA	36

NA: Question not asked in this year of the survey.

**Figure 4:**
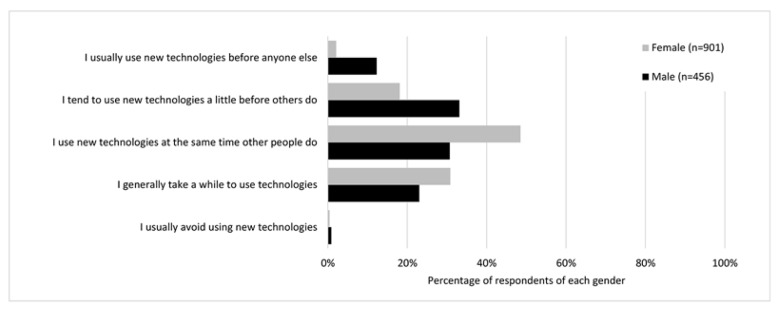
Technology adoption by gender

Respondents were also asked to characterize their technology adoption: 5.8% said they usually used new technologies before anyone else, 23.1% said they tended to use new technologies a little before others do, 42.5% said they used new technologies at the same time that other people do, 28.1% said they generally took a while to use new technologies, and 0.6% said they usually avoided using new technologies. Analysis of technology adoption by respondents’ gender showed males were significantly more likely to self-identify as early adopters than females (chi-square, *p*<0.01) (Figure 4).

**Figure 5:**
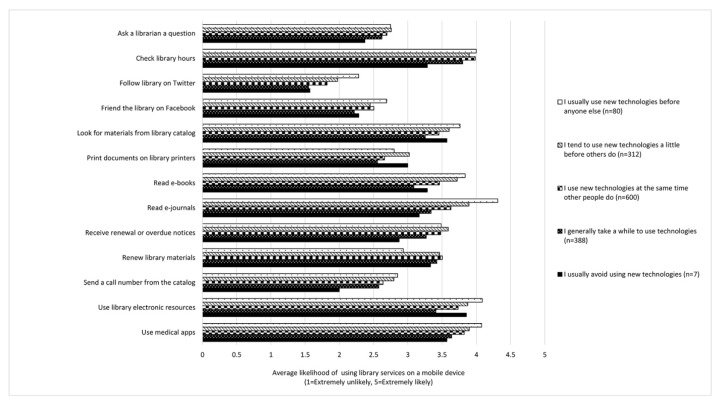
Likelihood of using library services on a mobile device by technology adoption

Likewise, respondents’ technology adoption was compared to their likelihood of using various library services on a mobile device. In most cases, early adopters were the most likely to use each library service, with each category of later adopters typically being less likely to use library services on their mobile devices. Figure 5 shows the results of this analysis for only library services that showed significant differences by technology adoption status.

## DISCUSSION

Many of the UF-specific responses over the last five years are similar to other implementations of the same survey, including Wu et al.’s aggregate data from five universities over one year [4] and data from the Spencer S. Eccles Heath Sciences Library at the University of Utah over two years [10]. For example, UF respondents indicated that they would be most likely to use the following services on their smartphones or tablets: check library hours, use medical apps, and use library electronic resources. These results are also similar in nature to those reported from the previous UF HSCL survey on smartphone usage and library support [1].

In response to these expressed user interests, the HSCL’s website was optimized to better highlight the library’s hours and to make these hours more prominent on the mobile version of the website. Given both the interest in medical apps reported in the current technology survey and the previously identified interest in a “menu of evaluated apps” [1], a “Mobile Resources for Health” LibGuide was launched in 2013. This LibGuide currently provides lists of clinical, administrative or productivity, research, instruction, patient education, and e-resources apps, with descriptions of content, platform, costs, and UF availability. The LibGuide also contains information on accessing UF-supported apps, as well as links to medical app reviews (from iMedical Apps) and advice on the safe and effective use of health apps from the American Medical Association.

Given that more than half of the respondents over the five years indicated that they would like training in the use of clinical apps, the significant decrease over time in the likelihood of using medical apps was a surprise to the team and was investigated further. A significant decrease in likelihood for this question occurred between 2015 and 2016; this decrease may be related to a change in the wording of the question from “use medical apps” to “use library-recommended apps.” The only other significant difference appeared when 2012 and 2015 were compared; it is unclear why these two years would differ significantly.

Le Ber et al. suggested that it was not surprising that the majority of students in their survey had not used bibliographic citation management tools, as first-year students had “not yet been required to manage large numbers of citations” [10]. Because the UF HSCL surveyed both new and returning students, it was possible to test this hypothesis in the UF environment. More new students than returning students had not heard of any of the citation management tools listed in the survey (EndNote [desktop], EndNote Web, Refworks, Zotero, Mendeley, Papers, BibMe), supporting this argument. While a fairly high percentage of UF respondents (students, faculty, and staff) indicated that they had never heard of or used EndNote tools, these two resources still scored more highly on the Likert scale than any of the other bibliographic citation resources that were included in the survey. This might reflect liaison librarians’ efforts to introduce EndNote during library orientations, course-integrated instruction, and individual consultations, as well as the EndNote stand-alone workshops that have been provided at least twice per semester.

Averaged over all 5 years of our survey, more UF HSCL respondents use tablets than their counterparts represented in the national survey [4] or those solely at Eccles [10]. This is the case even if only UF’s 2012 data are considered (since Wu’s report of the national survey only covers 2012 and the Eccles survey covers 2012 and 2013): UF, 51.1%; national, 34%; Eccles, <30%.

Extrapolation of the question related to tablet operating system showed that the percentage of UF respondents owning a tablet might be even higher. While it is unclear why UF faculty, students, and staff are heavy tablet users, direct evidence indicates that this is the case. In April of 2014, the UF HSCL made 30 iPads available for check-out. Although the iPads were not initially heavily promoted (while the library tested and fine-tuned its lending practices), the iPads are now widely advertised. Circulation rose from 137 in calendar year 2016 to 785 over the year from June 14, 2016, to June 14, 2017. Survey and circulation data emphasize the importance of understanding how these tablets are being used, as this usage has implications for collection development (app purchase), circulation policies, and development of new services.

In the area of user engagement through social media, the UF HSCL’s survey responses were similar to those reported in the previous tech surveys: averaged over the 5 years, most respondents reported being unlikely or extremely unlikely to friend the library on Facebook (59.7%) or follow the library on Twitter (80%). However, responses to the most recent iteration of the UF HSCL survey (2016) revealed that 25.3% (Facebook) and 11.1% (Twitter) of respondents were likely or extremely likely to do so. Additionally, there has been a significant increase over time in respondents’ likelihood of following the library on Twitter.

Although the percentages of respondents who are not interested in engaging with the library through social media are larger than those who expressed an interest, this positive component, if extrapolated to the entire user population of the HSCL, would be a sizable number of individuals. The marketing communications literature stresses the need for advertising in multiple modes for diverse user groups [11], such as those of the UF HSCL. Vucovich et al., following an exhaustive analysis of their health sciences library’s social media activities, describe instances when engaging users through social media can be successful. Facebook in particular was effective at marketing their library’s events and news [12].

While social media is one of the modes listed in the UF HSCL marketing communications plan, primarily for advertising events [13], the library has not had a coherent plan for routinely engaging users through social media until recent months. Based on the cohort who are interested in engaging with the library through social media, which was discovered through the UF HSCL survey, and successful applications in the literature, the UF HSCL recently developed a formal marketing team that created such a plan and is in the early stages of implementing it.

Like the results reported by Wu et al. [4] and Le Ber et al. [10], the results reported here showed that respondents to the UF HSCL survey preferred reading print over electronic. At UF, this preference was reported for both academic and leisure reading. These results corresponded to a national study of information-seeking behavior of natural science, engineering, and medical science academic researchers in which the UF HSCL and UF’s Marston Science Library participated [14]. In that study, the responding researchers preferred having both print and electronic versions of a resource available, suggesting that their preference varied based on type of use and information need. However, among those researchers who specifically preferred one format over the other, print won out at UF approximately 23% to 14%. Folb et al.’s study in clinical and academic medical settings reported that print was preferred for textbooks and manuals, while electronic format was preferred for research protocols and reference books [15], again suggesting that format preference can be task specific.

This preference for print, at least for some tasks, is a conundrum for institutions that are faced with the need for space (particularly study space) and budgets that cannot accommodate multiple formats. In the last 5 years, UF HSCL has removed 2 floors’ worth of print materials (a total of over 93,000 volumes) to accommodate renovations, with subsequent creation of new study and collaboration space. Despite preference for print, it is unlikely that the UF HSCL will be able to recover space to rebuild an extensive onsite print collection or increase its materials budget to allow purchases of multiple formats.

That change does not mean that the library should ignore the preferences for print, and understanding more about these preferences specifically at UF would be a fruitful area of research. Rethinking current policies regarding interlibrary loan (not ordering by format; not ordering if a resource is available at UF in any format) may be in order. Certainly, working with resource developers and vendors to make electronic resources more amenable to users—overcoming aversion to reading on the screen and issues related to difficulty in navigation and long-term preservation, among others—may facilitate a move toward electronic. Likewise, presenting more clearly to library users some of the benefits of electronic—24/7 access, availability for distance learners as well as students and residents on rotations, ability to read on multiple device types, annotation, searching, multiple individuals using a resource at once—may help mitigate some resistance to electronic resources.

The UF HSCL has a vibrant educational program, offering stand-alone workshops open to all several times a semester. In recent years, attendance has been relatively low in classes highlighting a specific bibliographic database (such as PubMed or Web of Science) and has been heaviest for more conceptual classes, such as those covering data management and citation management tools. Survey results suggest that users are interested in workshops on clinical medical apps, presentation tools, mobile device apps for research, and mobile device apps for productivity. In response, the HSCL has developed a sixty-minute stand-alone workshop encouraging attendees to think critically about and carefully evaluate new mobile resources. Workshop content parallels information found in the “Mobile Resources for Health” LibGuide described above, covering finding and evaluating apps, apps available through UF, and specialized apps (clinical, consumer health and patient education, research, teaching and learning, journals and books, productivity). The workshop was first offered in March of 2016 and has since been taught five additional times to a total of twenty participants.

Responses related to training interests were also analyzed by college. While no specific trends were discerned, respondents from the Colleges of Nursing and Dentistry showed the strongest interest in the top ten training opportunity categories (Nursing in six categories and Dentistry in four; Table 1). While all liaison librarians can use such data to inform their instructional planning, targeting the Colleges of Nursing and Dentistry for immediate instructional interventions may be an appropriate use of these data. In fact, because all data from the survey can be analyzed by college and by incoming versus returning students, it is expected that liaison librarians will find ways to use these data to customize both instruction and service provision.

The main limitation of this study is the low response rate (1.9%), which increases the potential for survey responses not being representative of the broader HSC population. The literature does indicate that survey response rates among health professionals are commonly under 20% and that paper surveys continue to have higher response rates than email surveys [16, 17]. Additionally, the response rate is based on an estimate of overall number of students, faculty, and staff at the 6 HSC colleges, averaging figures reported by the colleges in 2013 and 2016 and including a significant number of affiliate faculty in the count. These affiliate faculty, as well as staff, are not typically targets of librarian outreach and may not have been consistently included in the invitation to participate in the survey. Another potential limitation is volunteer bias, with those who took the survey perhaps doing so out of a particular interest in technology. This concern is somewhat mediated by participants’ perceptions of whether they are early, average, or late adopters of technology: responses to this question were slightly skewed toward early-adopters but, overall, representative of all levels of technology adoption.

While many of the UF HSCL results mirror findings in the literature, the annual review of survey results has allowed librarians to identify the local users’ needs and interests as they changed over time and has led to incremental changes in services offered. Reviewing the aggregate data allowed more strategic consideration of how technology impacts library interactions with these users, with implications toward marketing the library’s resources, training offered, and service development. Future work includes sharing the data more broadly across the HSCL, allowing liaison librarians to perform further analysis of results by college and adjust teaching and service provision accordingly. To maintain the capacity for longitudinal comparison, the survey questions have not changed significantly throughout the past five years. However, the authors plan to revise the survey significantly for 2017 to focus more specifically on the intersection of HSCL users, technology, and library services.

While the main benefit of this kind of user needs assessment to the HSCL is its specificity to the UF context, the results add to the body of literature on this topic and highlight trends in device ownership, use of library services, engagement with libraries on social media, and book format preferences that transcend a single institution.

## SUPPLEMENTAL FILES

Appendix A Survey instrument

Appendix B Summary of statistical results
