# Supplementary material for: Use of annual surveying to identify technology trends and improve service provision
Source: J Med Libr Assoc. 2018 Jul 1;106(3):320–9. doi: 10.5195/jmla.2018.324 (PMC6013140; doi:10.5195/jmla.2018.324)
Supplement: Appendix A [file jmla-106-320-s001.pdf]

JMLA Journal of the Medical Library Association

SC
Supplemental content to
J Med Libr Assoc. Jul;106(3):dx.doi.org/10.5195/jmla.2018.324
www.jmla.mlanet.org
© Norton, Tennant, Edwards, Pomputius 2018

Use of annual surveying to identify technology trends and improve service provision

Hannah F. Norton, MSIS, AHIP; Michele R. Tennant, PhD, MLIS, AHIP; Mary E. Edwards, MLIS, EdD, AHIP; Ariel Pomputius, MLIS

APPENDIX A

Survey instrument

Which of the following do you use? Check all that apply.

- Laptop computer (PC)
- Laptop computer (Mac)
- Desktop computer (PC)
- Desktop computer (Mac)
- Smartphone (iPhone, Android phone, Blackberry, etc.)
- Tablet (iPad, Asus Transformer, Sony Tablet S, Samsung Galaxy Tab, Windows, etc.)
- E-book reader (Kindle, Nook, Sony Reader, etc.)
- Wearable technology (Apple Watch, Samsung Gear, Garmin fitness watch, FitBit, etc.)
- Virtual reality hardware (Oculus Rift, HTC Vive, Google Cardboard, etc.)
- Other: ________________
- I don't use any of these

Which operating system (OS) do you have on your smartphone?

- iOS/Apple
- Google/Android
- Blackberry OS
- Windows Mobile
- Not sure
- I don't have a smartphone, but I'm planning to get one
- I'm not interested in any smartphone
- Other (please specify): _______________

Which operating system (OS) do you have on your tablet?

- iOS/Apple
- Google/Android
- Blackberry OS
- Windows Mobile
- Not sure
- I don't have a tablet, but I'm planning to get one
- I'm not interested in any tablet
- Other (please specify): _______________

Which brand of e-book reader do you use? Check all that apply.

- Kindle
- Sony
- Nook
- I use my laptop/desktop to read e-books
- I use my smartphone to read e-books

SC
Supplemental content to
J Med Libr Assoc. Jul;106(3):dx.doi.org/10.5195/jmla.2018.324
www.jmla.mlanet.org
© Norton, Tennant, Edwards, Pomputius 2018

- I use my tablet as an e-book reader
- I don't have an e-book reader, but I'm planning to get one
- I'm not interested in any e-book reader
- Other (please specify): _______________

For academic purposes, do you prefer reading e-books or print books?

- E-books
- Print books
- I don't have a preference

For leisure reading, do you prefer reading e-books or print books?

- E-books
- Print books
- I don't have a preference

If you own a smartphone/tablet, how likely would you be to use it for the following library services?

	Extremely unlikely	Unlikely	Fairly likely	Likely	Extremely likely
Look for materials from library catalog					
Use library electronic resources					
Use library-recommended apps					
Read e-books					
Read e-journals					
Check library hours					
Friend the library on Facebook					
Follow the library on Twitter					

If you own a cell phone, how likely would you be to use the following text/SMS library services?

	Extremely unlikely	Unlikely	Fairly likely	Likely	Extremely likely
Ask a librarian a question					
Send a call number from the catalog					
Receive renewal or overdue notices					

What, if any, other library resources/services you would like to use on your mobile device? [open response]

JMLA Journal of the Medical Library Association

SC
Supplemental content to
J Med Libr Assoc. Jul;106(3):dx.doi.org/10.5195/jmla.2018.324
www.jmla.mlanet.org
© Norton, Tennant, Edwards, Pomputius 2018

For each of these social sites, select the phrase that best describes your usage, including posting, reading, sharing, etc.

	Never heard of it	Never use it	Used it in the last year	Used it in the last month	Used it in the last week	Used it in the past 24 hours
Facebook						
FourSquare/Swarm						
Google+						
Instagram						
LinkedIn						
Pinterest						
Reddit						
Skype						
Snapchat						
Twitter						
Tumblr						
Vine						
YouTube						

For each of these citation tools, select the phrase that best describes your usage.

	Never heard of it	Never use it	Used it in the last year	Used it in the last month	Used it in the last week	Used it in the past 24 hours
EndNote (desktop)						
EndNote Web						
Mendeley						
Papers						
RefWorks						
Zotero						
BibMe						

Which of the following best describes you?

- I usually avoid using new technologies
- I generally take a while to use technologies
- I use technologies at the same time other people do
- I tend to use new technologies a little before others do
- I usually use new technologies before anyone else

JMLA Journal of the Medical Library Association

SC
Supplemental content to
J Med Libr Assoc. Jul;106(3):dx.doi.org/10.5195/jmla.2018.324
www.jmla.mlanet.org
© Norton, Tennant, Edwards, Pomputius 2018

The University of Florida (UF) Health Science Center Library currently offers training and classes on various topics (including some listed below), and we are considering expanding those options. Would you be interested in any of the following topics? Check all that apply.

- Clinical mobile device apps
- Mobile device apps for teaching and learning
- Mobile device apps for research
- Mobile device apps for productivity
- Patient-oriented mobile apps
- Finding and using academic e-books
- Google tools (Google Docs, Google Scholar)
- Photo editing tools
- Video editing tools
- Presentation tools (PowerPoint, Prezi)
- Citation tools (EndNote, RefWorks, Mendeley, etc.)
- Database searching (PubMed, Web of Science, etc.)
- Keeping up with current research
- 3D printing
- Virtual reality applications and programs
- Other (please specify): _______________

Additional comments [open response]

What is your status?

- Undergraduate student (BHS, BSN, etc.)
- Professional student (MD, PharmD, OT, PT, DDS, PA, etc.)
- Graduate student (Master, PhD, etc.)
- Resident
- Postdoctoral associate
- Faculty member
- Staff member
- Other (please specify): _______________

What is your affiliation? Check all that apply.

- College of Dentistry
- College of Medicine
- College of Nursing
- College of Pharmacy
- College of Public Health and Health Professions
- College of Veterinary Medicine
- Other (please specify): _______________

On which campus are you located? Check all that apply.

- Campus location: Gainesville
- Campus location: Jacksonville
- Campus location: Orlando
- Campus location: online/distance
- Other (please specify): _______________

JMLA Journal of the Medical Library Association

SC
Supplemental content to
J Med Libr Assoc. Jul;106(3):dx.doi.org/10.5195/jmla.2018.324
www.jmla.mlanet.org
© Norton, Tennant, Edwards, Pomputius 2018

Please select the age range which applies to you.
- 19 and under
- 20–25
- 26–30
- 31–35
- 36–45
- 46–55
- 56–65
- 66 and up

What is your gender?
- Male
- Female
- Prefer not to answer

What is your race/ethnicity? (Check all that apply)
- American Indian or Alaska Native
- Asian
- Black or African American
- Hispanic or Latino
- Native Hawaiian or other Pacific Islander
- White
- Other (please specify): _______________

If you are interested in providing future feedback on technology in the libraries, please click the link below. This will take you to a separate survey where you can enter your email address in order to be added to an informal advisory list. Your email address will not be linked to this Technology Survey in any way, and your responses to this Technology Survey will remain anonymous. [link]
