# Supplementary material for: Use of annual surveying to identify technology trends and improve service provision
Source: J Med Libr Assoc. 2018 Jul 1;106(3):320–9. doi: 10.5195/jmla.2018.324 (PMC6013140; doi:10.5195/jmla.2018.324)
Supplement: Appendix B [file jmla-106-320-s002.pdf]

JMLA Journal of the Medical Library Association

Supplemental content to
J Med Libr Assoc. Jul;106(3):dx.doi.org/10.5195/jmla.2018.324
www.jmla.mlanet.org
© Norton, Tennant, Edwards, Pomputius 2018

Use of annual surveying to identify technology trends and improve service provision

Hannah F. Norton, MSIS, AHIP; Michele R. Tennant, PhD, MLIS, AHIP; Mary E. Edwards, MLIS, EdD, AHIP; Ariel Pomputius, MLIS

APPENDIX B

Summary of statistical results

Question	Test used	Test value	Significance
Technology used by year	ANOVA		
Laptop computer (PC)		1.840	0.119
Laptop computer (Mac)		0.461	0.765
Smartphone		5.727	0.000*
Tablet		5.371	0.000*
E-book reader		1.873	0.113
Other		1.492	0.202
Smartphone operating system by year	ANOVA		
iOS		1.979	0.095
Google/Android		1.212	0.304
Blackberry		4.095	0.003*
Windows Mobile		0.315	0.868
Palm		0.989	0.397
Symbian		0.989	0.397
Not sure		5.336	0.000*
I don’t have a smartphone, but I’m planning to get one		8.755	0.000*
I’m not interested in any smartphone		4.611	0.001*
Other		2.153	0.072
Tablet operating system by year	ANOVA		
iOS		1.803	0.126
Blackberry		1.466	0.210
Google/Android		3.151	0.014*
Windows		2.840	0.023*
Not sure		0.897	0.465
I don’t have a tablet, but I’m planning to get one		1.498	0.201
I’m not interested in any tablet		1.953	0.099
Likelihood of using library services by year	ANOVA		
Look for materials from library catalog		1.115	0.348
Use library electronic resources		4.963	0.001*
Use medical apps		26.576	0.000*
Read e-books		2.133	0.075
Check library hours		3.256	0.011*
Friend the library on Facebook		1.688	0.150
Follow the library on Twitter		2.632	0.033*
Citation management tool use by year	ANOVA		
EndNote (desktop)		0.472	0.492
EndNote Web		3.732	0.054
RefWorks		0.310	0.578
Zotero		1.649	0.193
Mendeley		1.332	0.265
Papers		0.456	0.500
Any citation management tool use by new vs. returning student	chi-square	5.932	0.015*

JMLA Journal of the Medical Library Association

SC
Supplemental content to
J Med Libr Assoc. Jul;106(3):dx.doi.org/10.5195/jmla.2018.324
www.jmla.mlanet.org
© Norton, Tennant, Edwards, Pomputius 2018

Question	Test used	Test value	Significance
Academic book format preference by year	ANOVA		
Print books		0.599	0.550
E-books		0.429	0.652
No preference		0.635	0.530
Leisure book format preference by year	ANOVA		
Print books		0.232	0.793
E-books		0.635	0.530
No preference		0.164	0.849
Gender by technology adoption	chi-square	115.386	0.000*
Technology adoption by likelihood of using library services	chi-square		
Use medical apps		52.018	0.000*
Use library electronic resources		63.304	0.000*
Send a call number from the catalog		23.218	0.278
Renew library materials		21.464	0.370
Receive renewal or overdue notices		33.919	0.027*
Read e-journals		50.760	0.000*
Read e-books		100.105	0.000*
Print documents on library printers		10.102	0.813
Look for materials from library catalog		60.541	0.000*
Friend the library on Facebook		47.911	0.000*
Follow the library on Twitter		66.866	0.000*
Check library hours		34.187	0.025*
Ask a librarian a question		14.256	0.817

* Indicates statistical significance, $p<0.05$.
